# Intrahepatic cholangiocarcinoma coinciding with a liver metastasis from a rectal carcinoma: a case report

**DOI:** 10.1186/s40792-016-0222-x

**Published:** 2016-09-09

**Authors:** Shintaro Akabane, Masahiro Ohira, Tsuyoshi Kobayashi, Shintaro Kuroda, Naoki Tanimine, Seiichi Shimizu, Hiroyuki Tahara, Kentaro Ide, Kohei Ishiyama, Hiroyuki Egi, Kazuaki Tanabe, Kazuhiro Sentani, Wataru Yasui, Hideki Ohdan

**Affiliations:** 1 Department of Gastroenterological and Transplant Surgery, Applied Life Sciences, Institute of Biomedical & Health Sciences, Hiroshima University, 1-2-3, Kasumi, Minami-ku, Hiroshima 734-8551 Japan; 2 Department of Molecular Pathology, Institute of Biomedical & Health Sciences, Hiroshima University, Hiroshima, Japan

**Keywords:** Intrahepatic cholangiocarcinoma, Metastatic liver tumor, Colorectal carcinoma metastasis

## Abstract

**Background:**

We experience many cases of liver metastasis from colorectal cancer, but synchronous occurrence of intrahepatic cholangiocarcinoma (ICC) and liver metastasis from a rectal cancer is extremely rare. We herein report a case of ICC coinciding with a liver metastasis from a known rectal carcinoma.

**Case presentation:**

A 68-year-old man was referred to our hospital for investigation of multiple liver tumors. Total colonoscopy and computed tomography (CT) revealed a rectal carcinoma, coinciding with liver metastasis. He was planned to receive chemotherapy following rectal resection. During chemotherapy for the rectal cancer, one of the liver tumors gradually grew after first shrinking. The following hepatectomy revealed the presence of intrahepatic cholangiocarcinoma (ICC). Despite intensive chemotherapy for the ICC, he passed away 6 months after the hepatectomy.

**Conclusions:**

We should also suspect the possibility of multiple primary cancers, even if the patient has a history of cancer that is likely to cause metastatic lesions. When simultaneous neoplasms are diagnosed, systematic treatment should be targeted to the tumor with the worst prognosis.

## Background

The occurrence of multiple primary malignancies in a single organ is rare. There are several reports about synchronous double primary malignancies of the liver. The liver is the most frequent metastatic site of colorectal cancer, and therefore, hepatic masses of patients who have colorectal cancer are usually diagnosed as a metastasis of colorectal cancer. There was a case report of combined hepatocellular carcinoma-cholangiocarcinoma harboring a metastasis of colon adenocarcinoma that was treated with curable resection [1]. However, in our case, one of several liver tumors gradually developed during chemotherapy for the known rectal cancer. Hepatectomy and pathology revealed it was an intrahepatic cholangiocarcinoma (ICC), not a metastasis from a rectal cancer. To our knowledge, this is the first case report of such a simultaneous condition.

## Case presentation

A 68-year-old man was referred to our hospital for investigation of multiple liver tumors. He had a history of drinking shochu (60 g of alcohol per day). His medical history was unremarkable with the exception of hypertension. Serum biochemistry was as follows: aspartate aminotransferase, 19 U/L; alanine aminotransferase, 19 U/L; total bilirubin, 0.1 mg/dL; carcinoembryonic antigen (CEA), 6.0 ng/mL; cancer antigen 19-9 (CA 19-9), 2601 U/mL; alkaline phosphatase, 183 U/L; gamma-glutamyl transpeptidase, 36 U/L. He was negative for hepatitis B antigen and hepatitis C antibodies.

**Fig. 1:**
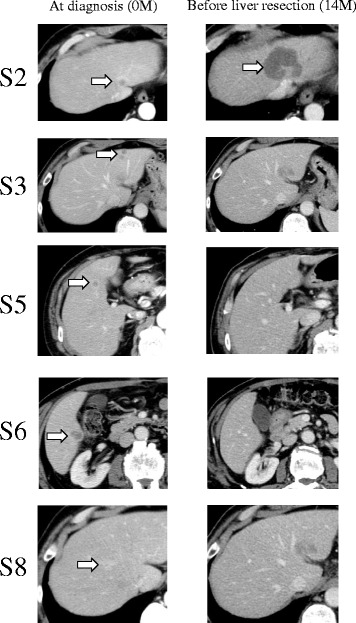
Computed tomography (CT) showed an enhanced mass in the rectum and hypo-vascularized tumors in the liver segment 2 (S2), segment 3 (S3), segment 5 (S5), segment 6 (S6), and segment 8 (S8). The largest liver tumor had a diameter of 16 mm in S2. Almost all of the multiple liver tumors had shrunk after the neoadjuvant chemotherapy, but the S2 tumor had grown on the CT scan (16 to 46 mm)

Computed tomography (CT) showed an enhanced mass in the rectum and hypo-vascularized tumors in the liver segment 2 (S2), segment 3 (S3), segment 5 (S5), segment 6 (S6), and segment 8 (S8) (Fig. 1). The largest liver tumor had a diameter of 16 mm in S2. On positron emission tomography-computed tomography (PET-CT), abnormal fluorodeoxyglucose (FDG) uptake was seen at the rectum (SUV-max 12.7) and the S2, S5, and S8 tumors (SUV-max 10.2). Total colonoscopy revealed a rectal carcinoma with a diameter of 3 cm that was located 10 cm distant from the anal verge.

He was diagnosed with rectal carcinoma coinciding with liver metastasis. His preoperative staging was T4N1M1 stage IV (TNM classification). Numerous metastatic tumors already occupied in the liver. We estimated that 61 % of the whole liver needed to be resected for the complete excision. Therefore, he was planned to receive chemotherapy following rectal resection to avoid liver failure. Laparoscopic lower anterior resection was performed, and pathological examination showed moderately differentiated adenocarcinoma. According to the TNM classification, pathological staging was diagnosed as T3N2M1 (stage IV). The postoperative course was uneventful, and he was discharged on the eighth postoperative day.

The 10 courses of chemotherapy with capecitabine and oxaliplatin (XELOX) plus bevacizumab (Bmab) started a month after the operation and continued for 8 months. His tumor markers and liver mass decreased, but after a short time, his tumor markers elevated again (CEA 9.5 ng/mL, CA19-9 9027 U/mL). Therefore, we changed the regimen to xeloda, irinotecan, and bevacizumab (XELIRI + Bmab) and administered four courses. Although almost all of the multiple liver tumors had shrunk, the S2 tumor had grown as seen on a CT scan (16 to 46 mm) and had FDG increased uptake (SUV-max 11.9) on PET-CT. There were no signs of local, distant, or lymph node metastases.

**Fig. 2:**
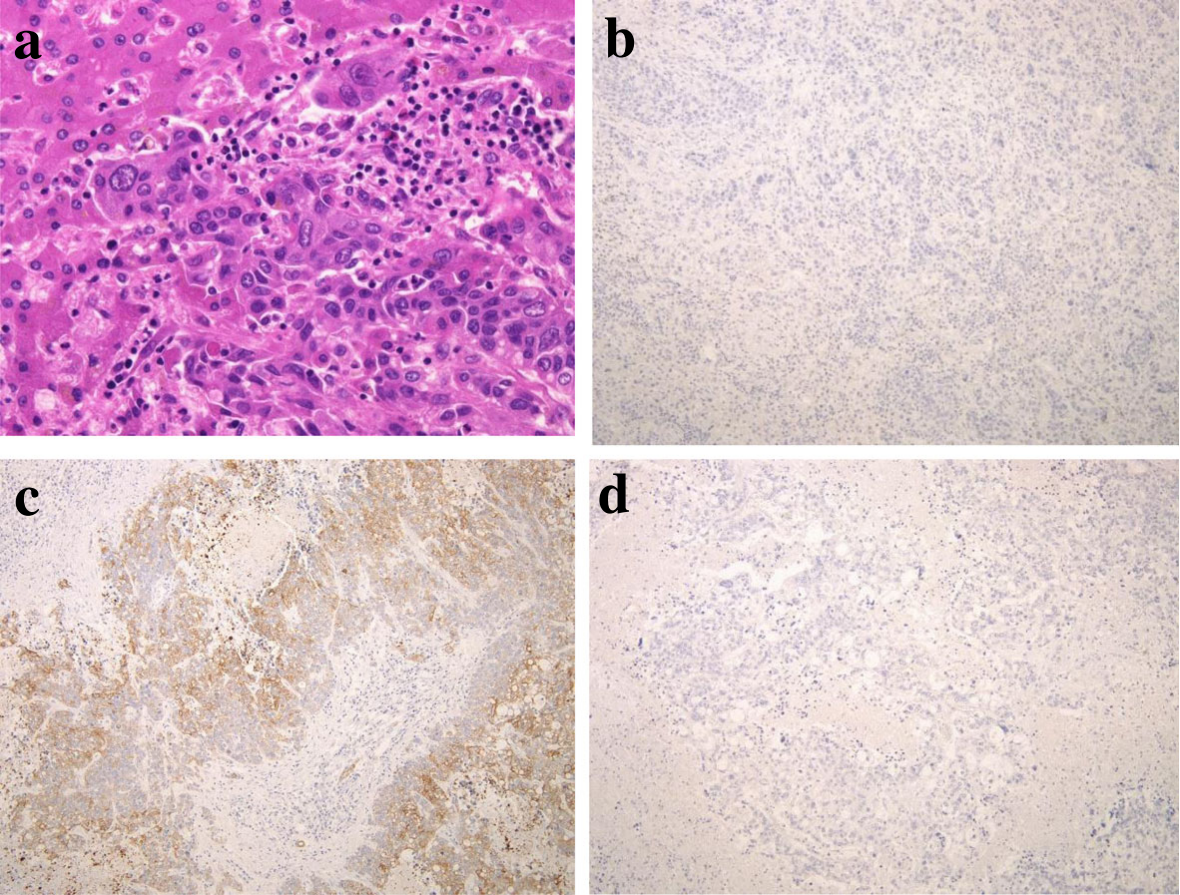
**a** Microscopic findings of the S2 tumor show moderately differentiated intrahepatic cholangiocarcinoma (H&E staining ×400). **b** Carcinoma cells are negative for CDX-2 (on immunohistochemistry, ×100). **c** Carcinoma cells are positive for CD19 (on immunohistochemistry, ×100). **d** Carcinoma cells are positive for hepatocytes (on immunohistochemistry, ×100)

We assumed the neoadjuvant chemotherapy was not effective for the S2 lesion, so we decided to perform an extended left hepatectomy. The images of the tumor are shown in Fig. 1, and the clinical course is summarized in Table 1. To our surprise, pathological examination showed intrahepatic cholangiocarcinoma (ICC), moderately differentiated, 55 mm × 50 mm × 40 mm, s1, ne1, vp1, vv1, va0, b1. The carcinoma cells were positive for CD10 and CD19 and negative for hepatocyte, glypican 3, CK7, and CDX2 (Fig. 2). In the segments of hepatic tissue, no metastatic adenocarcinoma was found. According to the TNM classification, the pathological stage of the ICC was IVa (T4N0M0). The postoperative course was uneventful, and he was discharged on the 12th postoperative day.

**Table 1:** Clinical course, tumor size, and serum tumor markers

Months	Clinical course	Tumor size (CT) (mm)					Tumor marker		Evaluate the response to treatment
		S2	S3	S5	S6	S8	CEA (ng/mL)	CA19-9 (U/mL)	
0	At diagnosis	16	12	12	7	8	6	2601	
1	Lower anterior rectal resection								
2	XELOX + Bmab (1st course)						45.9	22,751	
5	XELOX + Bmab (4th course)	18	0	0	0	0	5.4	515	PR
7	XELOX + Bmab (7th course)						4.4	166	
9	XELOX + Bmab (9th course)						4.4	873	
11	XELIRI + Bmab (1st course)	21	0	0	0	0	9.5	9027	SD
13	XELIRI + Bmab (4th course)						43.4	42,380	PD
14	Extended left hepatectomy	46	0	0	0	0			
16	Gemcitabine + TS-1						10.9	2345	

Clinical course and data are summarized. As CA 19-9 level had been increased during the XELOX + Bmab therapy, we changed the regimen to XELIRI + Bmab

A month later, PET-CT showed metastasis to the peritoneum, lumbar vertebra, lung, and skin. Biopsy of the skin revealed a metastasis of cholangiocarcinoma. Despite intensive chemotherapy with gemcitabine and TS-1 (GS), and then gemcitabine and cisplatin (GC), he passed away 6 months after the hepatectomy. An autopsy was not performed at the request of the family.

### Discussion

The main finding of the present case is that ICC unexpectedly existed in the liver among metastatic tumors from colon cancer. Liver resection was scheduled after the planned chemotherapy ended. However, contrary to our expectation, ICC incidentally developed during the chemotherapy for rectal cancer. Our case is instructive of the need to pay attention to the possibility of multiple primary cancers in that situation.

ICC is a relatively rare type of primary liver cancer, which accounts for only 5–10 % of liver malignancies [2]. Due to the increased life expectancy and improved screening programs, the early detection ratio for multiple primary malignancies is expected to increase [3]. Synchronous cancers are defined as those where secondary tumors occur simultaneously or within 6 months of diagnosis of the primary malignancy [4]. ICC sometimes coexists with other cancers such as HCC [5–8], GIST [9, 10], thyroid cancer [11], lymphoma [12], and renal cell carcinoma [13]. Only one case with synchronous double cancer consisting of combined HCC-ICC and metastasis of a colon adenocarcinoma, which were treated by curable resection, has been reported [1].

In our case, it was difficult to diagnose the ICC before hepatectomy because the CT scan showed that there were several tumors in the liver that were similar in shape. Nam et al. reported a case of advanced synchronous GIST and ICC, which was operable at initial presentation, but progressed to become surgically unresectable [9]. They concluded that the possibility of multiple primary tumors must be considered as an alternative diagnosis.

In our case, the chemotherapy for colorectal cancer was continued for 8 months because only the S2 site tumor remained. ICC has been shown to be resistant to common chemotherapy, with an unacceptably low response rate [14]. Double gemcitabine and cisplatin therapy is currently considered the first-line therapy for patients with advanced disease [15, 16].

As we have shown in Table 1, CA 19-9 level had been increased during the XELOX + Bmab therapy, we should have taken into account the operation instead of changing the regimen to XELIRI + Bmab. If the S2 tumor was resected earlier, this patient might have better prognosis. Pintea et al. concluded that when simultaneous neoplasms are diagnosed, systematic treatment can be performed and this should target the tumor with the worst prognosis [1]. We believe this is a suggestive case in which we needed to take into account the possibility of multiple primary tumors.

## Conclusions

We experienced an instructive case from which we learned to suspect the possibility of a cholangiocarcinoma when we detect a solid lesion in the liver of a patient who has colorectal cancer. We should also suspect the possibility of multiple primary cancers, even if the patient has a history of cancer that is likely to cause metastatic lesions. We need to try for an accurate diagnosis, and when simultaneous neoplasms are diagnosed, systematic treatment should be targeted to the tumor with the worst prognosis.

## Consent

Written informed consent was obtained from the patient for publication of this case report and any accompanying images. A copy of the written consent is available for review by the Editor-in-Chief of this journal.

## Abbreviations

CA 19-9: Cancer antigen 19-9
CEA: Carcinoembryonic antigen
CT: Computed tomography
FDG: Fluorodeoxyglucose
GIST: Gastrointestinal stromal tumor
H&E: Hematoxylin and eosin
ICC: Intrahepatic cholangiocarcinoma
MRI: Magnetic resonance imaging
PET: Positron emission tomography
SUV: Standardized uptake value

## References

[1] Pintea B, Di Tommaso L, Destro A, Roncalli M (2013). Combined hepatocellular carcinoma-cholangiocarcinoma harboring a metastasis of colon adenocarcinoma. J Gastrointestin Liver Dis.

[2] Tsai S, Nathan H, Pawlik TM (2010). Primary liver cancer: intrahepatic cholangiocarcinoma emerges from the shadows. Updat Surg.

[3] Moertel CG (1964). Incidence and significance of multiple primary malignant neoplasms. Ann N Y Acad Sci.

[4] Moertel CG (1977). Multiple primary malignant neoplasms. Hist Perspect Cancer.

[5] Fuji N, Taniguchi H, Amaike H, Oka K, Tsuchihashi Y, Urasaki K (2005). Synchronously resected double primary hepatic cancer, hepatocellular carcinoma and cholangiocarcinoma. J Gastroenterol Hepatol.

[6] Inaba K, Suzuki S, Sakaguchi T, Kobayasi Y, Takehara Y, Miura K (2007). Double primary liver cancer (intrahepatic cholangiocarcinoma and hepatocellular carcinoma) in a patient with hepatitis C virus‐related cirrhosis. J Hepato-Biliary-Pancreat Surg.

[7] Geramizadeh B, Gity R, Bahraini A, Malek-Hosseini S (2014). Synchronous hepatocellular carcinoma and cholangiocarcinoma in a patient transplanted for cryptogenic cirrhosis. Int J Organ Transplant Med.

[8] Wu C, Bai D-S, Jiang G-Q, Jin S-J (2014). Synchronous double cancers of primary hepatocellular carcinoma and intrahepatic cholangiocarcinoma: a case report and review of the literature. World J Surg Oncol.

[9] Nam SJ, Choi HS, Kim ES, Keum B, Jeen YT, Chun HJ (2015). Synchronous occurrence of gastrointestinal stromal tumor and intrahepatic cholangiocarcinoma: a case report. Oncol Lett.

[10] Lao X-M, Ye Z-Y, Guo R-P, Guo Z-X, Wang G-H, Li J-Q (2009). A gastrointestinal stromal tumor of the jejunum associated with intrahepatic cholangiocarcinoma and pulmonary hamartoma: a case report. Acta Oncol.

[11] Wang QL, Li XJ, Zhao K, Liu BO, Ye XM (2015). Synchronous double primary cancer—intrahepatic cholangiocarcinoma with bone metastases and thyroid carcinoma: a case report. Oncol Lett.

[12] Fwu CW, Chien YC, You SL, Nelson KE, Kirk GD, Kuo HS (2011). Hepatitis B virus infection and risk of intrahepatic cholangiocarcinoma and non-Hodgkin lymphoma: a cohort study of parous women in Taiwan. Hepatology.

[13] Levy BF, Nisar A, Karanjia ND (2006). Cholangiocarcinoma, renal cell carcinoma and parathyroid adenoma found synchronously in a patient on long-term methotrexate. HPB (Oxford).

[14] Furuse J, Kasuga A, Takasu A, Kitamura H, Nagashima F (2012). Role of chemotherapy in treatments for biliary tract cancer. J Hepatobiliary Pancreat Sci.

[15] Valle J, Wasan H, Palmer DH, Cunningham D, Anthoney A, Maraveyas A (2010). Cisplatin plus gemcitabine versus gemcitabine for biliary tract cancer. N Engl J Med.

[16] Maithel SK, Gamblin TC, Kamel I, Corona‐Villalobos CP, Thomas M, Pawlik TM (2013). Multidisciplinary approaches to intrahepatic cholangiocarcinoma. Cancer.

